# Recall of Psychotherapy Information and Immersiveness in a Virtual Reality Application for Cancer-Related Chronic Pain

**DOI:** 10.1089/jmxr.2024.0019

**Published:** 2024-11-06

**Authors:** Alwin Chuan, Melissa Hatty, Mike Shelley, Angus Wilson, Angela Lan, Anton Bogdanovych

**Affiliations:** 1 South Western Sydney Clinical Campus, Faculty of Medicine and Health, University of NSW, Sydney, Australia.; 2 BehaviourWorks Australia, Monash Sustainable Development Institute, Monash University, Clayton, Australia.; 3 Northern Integrated Pain Management, Newcastle, Australia.; 4 MARCS Institute for Brain, Behaviour and Development, Western Sydney University, Sydney, Australia.

**Keywords:** virtual reality, cancer, chronic pain, education, immersive

## Abstract

**Introduction:**

There is increasing interest in the use of virtual reality (VR) as a modality to provide health interventions to patients. An unmet need in patients with cancer-related chronic pain is the availability of effective nondrug, pain psychology-based therapies that improve function and quality of life. To address this need, we previously created a custom-made VR program that teaches how to self-administer progressive muscle relaxation and guided pain visualization therapy in patients with cancer. We sought to determine if knowledge retrieval of these therapies is affected when comparing the same content delivered via a VR head-mounted display and hand motion controllers (“VR group”) or via 2D monitor and keyboard/mouse on a traditional personal computer (“PC Monitor group”).

**Methods:**

Randomized controlled study of healthy volunteers divided into VR group and Monitor group. Primary outcome was multiple-choice question scores between groups at two time periods (immediately after use and 2 weeks follow-up) testing knowledge recall of the therapies. Secondary outcomes were levels of immersion and satisfaction.

**Results:**

Forty healthy adults were recruited. Participants rated the VR-based version as highly immersive compared with the PC Monitor version (*p* < 0.001). There was no difference in knowledge retrieval both between groups (*p* = 0.98) and within groups (*p* = 0.58) after adjusting for age, education, and video game experience. Both the VR and PC groups had no quantitative difference in satisfaction (*p* values all >0.06) but qualitatively preferred the VR for clinical use despite more feedback on cybersickness side effects.

**Conclusion:**

This study provided further evidence on how to implement VR in clinical settings to improve acceptability and equivalency to traditional methods of chronic pain education.

## Introduction

Cancer can cause pain in patients due to the growth of the tumor, surgery for cancer, and from side effects of chemotherapy and radiation therapy. Unfortunately, chronic pain is reported by up to 75% of patients with cancer, with that pain suboptimally managed in 43% of patients.^1^ One important component of a multimodal approach to cancer-related chronic pain is the use of nondrug, pain psychological therapies that aim to improve cognitive and emotional adaption.^2^ The best evidenced pain psychological therapies for cancer pain are progressive muscle relaxation and guided pain visualization.^3,4^ The former is a type of structured arousal reduction therapy that aims to reduce muscle spasms and tension, while the latter is a cognitive-behavioral therapy that aims to reduce pain catastrophizing and improve the functional quality of life.

Virtual reality (VR) has been used to deliver analgesia to patients with cancer, with earliest studies showing promise in pediatric and adult patients with cancer.^5,6^ However, our group showed in a recent systematic review that the overall quality of evidence is poor, with most studies using VR as simple distractions (passively watching pre-recorded videos) to manage acute cancer pain and anxiety.^7^ We hypothesized that immersive VR, with its ability to create life-like and highly distracting user experiences, is important by its ability to divert attention away from the acute pain stimulus, but much less evidence is available for management of chronic pain.

There, thus, exists a need to research the utility of VR when used for chronic cancer pain and to incorporate the more sophisticated psychological therapies established for cancer use. Furthermore, it is unknown if quality of VR immersion is as necessary given that education, rather than distraction, is the goal of these psychological therapies.

We recently created a custom-made VR software that incorporated both progressive muscle relaxation and guided pain visualization therapies to manage chronic pain in patients with cancer (henceforth, “VR program”).^8,9^ While full details are in our previous publications, we provide the following description: Our research team pain psychologists (authors M.H. and M.S.) converted their face-to-face progressive muscle relaxation and guided pain visualization therapies into VR format with the help of our computer scientist (author A.B.). Captivating scenery (high-resolution jungle and beach scenes), interactive avatars (anatomically correct human model for muscle relaxation and angry pain character for guided pain visualization), and soundtrack were programmed to create an immersive visual and auditory virtual environment. We also purposefully designed the VR program to reduce the risk of cybersickness, which manifests as symptoms of nausea, disorientation, dizziness, headaches, and eyestrain when using electronic devices. We did this by limiting head and arm movements (the interactions within the VR environment are restricted to directly ahead of the user’s field of view), minimizing rapid eye tracking (VR characters do not move quickly across the field of view), and reducing disorientation (avoiding sudden scene changes or transitions).

To ensure fit-for-purpose, we recently performed a randomized controlled trial recruiting patients with cancer with chronic neuropathic pain, with primary outcomes of feasibility (ability to recruit appropriate patients, low withdrawal rates, and to collect high-quality outcomes data) and acceptability (patients can tolerate wearing the VR headset with minimal cybersickness). Over 2 weeks, one group of patients used our VR program to learn psychological therapies to help manage their chronic pain versus another group who received VR distraction therapy. At 3-month follow-up, we showed that patients using our VR program experienced reduced cybersickness and greater acceptability of VR-based interventions.^9^ This was an important first step in validating our VR program for clinical use. There was also a trend toward reductions in opioid use and pain scores, but this was statistically not significant, and our trial was not powered for these secondary outcomes.

In this randomized controlled study, we sought to continue the process of validating the VR program for clinical use, by answering the following questions: (1) How immersive is our program’s virtual environment when experienced via a head-mounted display and hand motion controllers (“VR group”) as compared with a 2D monitor and traditional personal computer (“PC Monitor group”)? (2) Does higher immersion improve knowledge retrieval of the pain psychological therapies taught by our VR program, especially since previous studies on educational recall of VR-delivered information give contradictory results? (3) How satisfied are users of the VR program for its intended clinical application: would they use it themselves and recommend it to other patients? Results from this study will assist in deeper understanding of the characteristics of the VR program as well as its limitations and to help plan future clinical trials that use VR for therapeutic applications in chronic cancer pain.

## Materials and Methods

### Ethics approval and participants

This prospective, single center, randomized controlled trial was approved by the South West Sydney Local Health District Human Research Ethics Committee (2020/STE03803) and prospectively registered in the Australian and New Zealand Clinical Trials Registry (ACTRN12620001087943). Eligible participants were healthy adults aged at least 18 years old, not currently experiencing acute or chronic pain, not receiving medications or under therapy for psychological or psychiatric conditions, have written and verbal English language proficiency, able to be followed up over 2 weeks, and never had exposure to pain psychological therapies including guided pain visualization or progressive muscle relaxation. We recruited healthy volunteers and chose a convenience sample size of 40 participants as the primary aims were to test immersion between the VR and 2D versions of the program and to test information recall without distractions such as co-existing painful conditions and opioid medications that would confound the outcome. There were no other exclusion criteria. As a volunteer study, we provided advertisements and the study protocol to members of the Department of Anaesthesia at Liverpool Hospital, Sydney. Staff, their families, and their friends were invited to read the advertisement and to approach research staff if they wished to participate. After checking for eligibility and explanation of the study protocol, informed consent was obtained and participants were recruited. No financial incentives were provided for participation.

### Procedure

All participants (*n* = 40) were randomized to VR (*n* = 20) or PC Monitor-based (*n* = 20) groups by a computer-generated, sex-stratified, 1:1 allocation table. Our VR setup was a Core i7-8750H gaming laptop (Intel Corp, Mountain View, CA, USA) with GeForce GTX 1060 graphics core (NVIDIA Corp, Santa Clara, CA, USA) running an Oculus Rift S (Meta Platforms, Menlo Park, CA, USA) and hand-held controllers. The PC Monitor setup was the same laptop running the pain therapy program on an external monitor with interactions made using a computer keyboard and mouse.

Participants were brought singly into a secluded quiet room without other distractions (e.g., no background music) and with only the one research assistant present. They were seated in a chair in front of a desk with the VR setup and laptop ready to be used. Participants in the VR group received a single 30-min exposure to the VR program, which consisted of a 15-min session of guided pain visualization followed immediately by a 15-mi session of progressive muscle relaxation therapies. Participants interacted with avatars in the virtual environment using their hand-held motion controllers. Participants in the PC Monitor group received the same 30-min exposure to the pain therapy program but displayed on a conventional 2D monitor.

### Timepoints of data collection

**FIG. 1.**
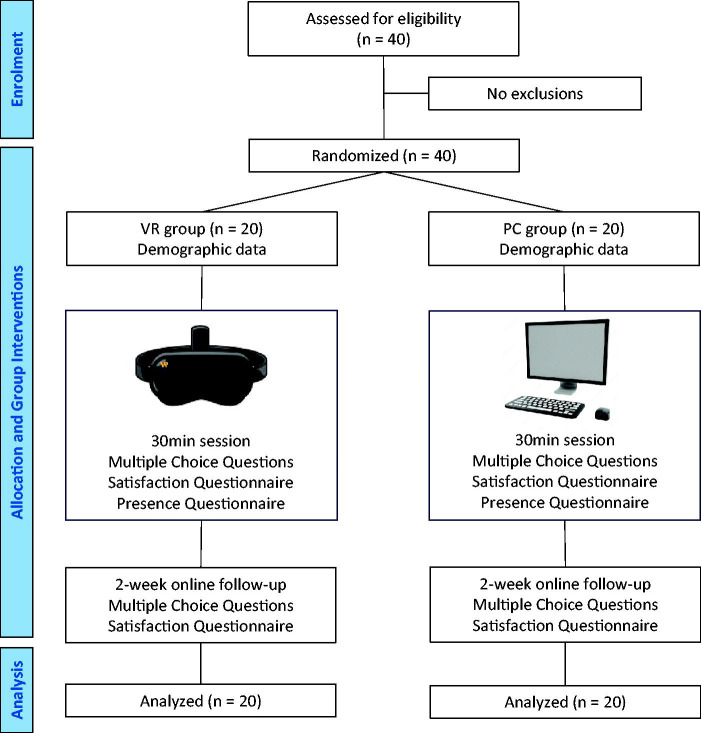
CONSORT flow diagram of study.

**FIG. 2.**
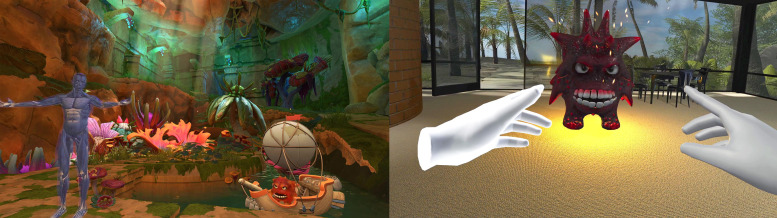
Screenshots of our progressive muscle relaxation therapy (left) and guided pain visualization therapy (right) that are included in the VR program. VR, virtual reality.

The CONSORT flow diagram illustrating the data collection and group allocations is shown in Figure 1. Prior to using the pain therapy program, all participants provided data on age, sex, total years (either full or parttime) of tertiary-level education after secondary school and number of years of playing computer games (defined as regularly playing at least 1 gaming session per week, in either desktop- or VR-based computer games). Immediately after using the program, all participants completed a multiple-choice question (MCQ) test comprising 10 stem questions, each with 4 options (1 correct and 3 distractor options). The MCQs tested knowledge retrieval on critical facts on chronic pain, and the most important factual aspects of progressive muscle relaxation and guided visualization as taught in the VR program (Fig. 2). No feedback was provided to participants after completing the MCQs. These MCQs are not part of the normal VR program and were constructed specifically for this study by the research team pain psychologists (authors M.H., M.S.) who originally designed the VR program.

All participants also completed a satisfaction questionnaire, comprising three questions that participants scored by marking their answer on a visual analog scale (0–10). The questions were Question 1 “After completing this teaching method for pain therapy, how confident are you in doing these exercises by yourself?” (0 = not at all confident, 10 = very confident); Question 2 “How likely would you use this teaching method for pain control?” (0 = not likely at all, 10 = very likely); and Question 3 “How likely are you to share this teaching method for pain therapy to a relative or friend? (0 = not likely at all, 10 = very likely). A free text section allowed participants to provide other feedback. Finally, all participants completed the Witmer and Singer Presence Questionnaire (version 3, Université du Québec en Outaouais Cyberpsychology Laboratory, Canada), to measure the immersiveness of the VR- and PC Monitor-based content.^10^

After a fortnight, all participants were automatically provided with an email reminder and electronic link to complete the MCQ and satisfaction questionnaire again. To prevent recall bias, the MCQ stems were randomized in presenting order, and their stem options were also randomized. The satisfaction questionnaire was unchanged. The MCQ and satisfaction questionnaires were hosted online on a REDCap server to ensure confidentiality of answers, with each participant receiving a unique link to complete the survey. The MCQ and satisfaction surveys are included in Supplementary Data S1.

### Statistical analysis

The primary outcome was MCQ scores between groups over both time periods (immediately after and fortnight after completing the pain therapy program). Factorial repeated measures analysis of variance was used to compare MCQ scores using Wilk’s lambda, adjusted with the following co-variates: age, educational attainment, and number of years of gaming. The 22-item Presence Questionnaire was analyzed between groups using *t*-tests, comparing the predefined six domains (a seventh domain on haptics was invalid as haptic feedback is not programmed) and total score. Raw scores were then converted to percentage scores of maximum (0% = totally unlike real life, 100% = exactly like real life). The quantitative answers for the satisfaction questionnaires were analyzed using Mann–Whitney tests. All analyses were performed using SPSS Statistics (version 24, IBM Corp, Armonk, NY, USA), with two-tailed significance *p* < 0.05.

## Results

The demographic data of the 40 recruited participants are shown in Table 1. All participants completed the study and answered all questionnaires. Immersiveness of the pain therapy in the virtual environment was significantly higher compared with when viewed on a PC Monitor, both in total (*p* < 0.001) and in all domains (*p* < 0.004) with the exception of quality of interface (*p* = 0.50) (Table 1). There was no difference in knowledge retrieval between VR-delivered and PC Monitor-delivered pain therapy programs across both time periods (*p* = 0.98), nor was there any difference over time within both groups (*p* = 0.58) after adjusting for age, education, and video game experience (Table 2). There was no difference between groups for satisfaction (*p* values 0.06–0.99) (Table 3). Ten text feedback were received from VR group participants, including four comments on cybersickness (“got a bit dizzy”, “nausea”) and the VR headset (“claustrophobia”); positive comments included “great experience,” “beneficial,” and “good for relaxing muscles.” Eleven text feedback were received from PC group participants, including five comments on perceived ineffectiveness (“very annoying,” “boring,” “couldn’t move around,” “don’t think these techniques are effective,” “interactive method would have been better”); positive comments included “good on the whole,” “a logical adjunct to pain management,” and “overall the techniques are good.” Verbatim comments are included in the Supplementary Data S1

**Table 1.** Demographics of Participants and Immersiveness of the VR Pain Therapy Program When Used with a VR Head-Mounted Display and Hand Motion Controllers (VR Group) and on a Traditional Computer Keyboard and Mouse with 2D Monitor (PC Monitor Group) as Measured by the Presence Questionnaire

	Total (*n* = 40)	VR group (*n* = 20)	PC Monitor group (*n* = 20)	*p*-Value
Sex (Female/Male)	20/20	10/10	10/10	1.0
Age (years)	44 (24–51) [18–70]	47 (35–52) [18–70]	41 (23–51) [21–68]	0.50
Years of tertiary education (years)	4 (2–5) [0–11]	4 (2–5) [0–11]	4 (2–5) [0–10]	0.90
Video game use (years)	0 (0–3) [0–30]	0 (0–1) [0–10]	0 (0–9) [0–30]	0.74
Presence Questionnaire				
(raw scores, %)				
Realism		37 (75%)	21 (43%)	<0.001
Possibility to act		21 (77%)	10 (36%)	<0.001
Quality of interface		16 (78%)	16 (75%)	0.50
Possibility to examine		15 (71%)	9 (43%)	<0.001
Self-evaluation		12 (84%)	6 (45%)	<0.001
Sound		17 (83%)	14 (66%)	0.004
Total		119 (77%)	76 (49%)	<0.001

Continuous values reported as median (interquartile range) [range].

PC, personal computer; VR, virtual reality.

**Table 2.** Multiple-Choice Question Scores by Groups, Immediately and Fortnight After Using the VR Pain Therapy Program by the VR and PC Monitor Groups

	VR group	PC Monitor group	Between groups performance *p*-value
MCQ Test 1 (immediate)	80 (70–97) [30–100]	90 (72–100) [30–100]	0.98
MCQ Test 2 (fortnight)	85 (70–90) [40–100]	90 (72–100) [20–100]	
Within-group performance over time *p*-value	0.58		

**Table 3.** Satisfaction Questionnaire Scores by Group, Immediately and Fortnight After Using the VR Pain Therapy Program by the VR and PC Monitor Groups

	VR group	PC Monitor group	Between groups *p*-value
Satisfaction Test 1 (immediate)			
Q1. Confidence in using therapy	9 (8–10) [6–10]	9 (7–10) [2–10]	0.68
Q2. Using therapy for pain relief	8 (6–9) [6–9]	7 (5–9) [0–10]	0.99
Q3. Sharing therapy to others	8 (7–9) [5–10]	7 (5–9) [0–10]	0.45
Satisfaction Test 2 (fortnight)			
Q1. Confidence in using therapy	8 (7–9) [5–9]	7 (6–8) [3–10]	0.06
Q2. Using therapy for pain relief	8 (7–9) [6–10]	7 (6–8) [0–10]	0.16
Q3. Sharing therapy to others	8 (6–9) [5–10]	7 (6–8) [0–10]	0.57

Continuous values reported as median (interquartile range) [range].

## Discussion

In this randomized controlled trial, participants found the VR program to be highly immersive and closer to real life, when compared with the same program displayed on a 2D computer monitor. However, this increased immersion did not lead to significant differences in knowledge retrieval between groups. There were qualitatively more feedback on perceived usefulness of the VR-based program, but equally there were feedback on VR-specific disadvantages including cybersickness and headset discomfort.

Educating patients in pain management is an essential component of treating cancer-related chronic pain, even though it is still unclear which delivery method (individual, group-based, written, electronic, duration, and follow-up) is best.^11^ With more widespread availability of VR devices, it is now possible to deliver pain psychological therapies in a highly immersive, multimodal manner that engages a patient visually, audibly, and interactively.

In this study, we found that immersiveness is not associated with better factual learning. Published literature has found both a positive association,^12–14^ as well as a negative association,^15–17^ of VR immersiveness and knowledge retrieval. Hypotheses to explain reduced association are based on the concept of cognitive load, with the vividness and novelty of virtual environments paradoxically distracting the user so much that working memory is impaired.^15,18^ Similarly, higher levels of emotional arousal due to more intense VR experiences may impede cued recall especially if the content is upsetting.^16^ In another study, the style of presentation of medical information (displayed as a floating text box versus the same content displayed as a textbook) changes the degree of knowledge retrieval, while audio tracks may enhance or diminish recall depending on the information type.^19^ Taken together, these studies justify our protocol and points to the need to check the validity of newly developed educational content.

A major consideration when designing our VR program for patients with cancer minimizing cybersickness,^20^ as this population is at higher risk due to a combination of both their cancer and to side effects from chemotherapy. Despite this, we found in our previous article^9^ that VR-associated cybersickness occurred in 20–40% of patients with cancer. Importantly, the rate of cybersickness was similar between the control group (used VR to watch documentary videos) and the intervention group (used the VR program), suggesting that the VR program did not increase the risk of cybersickness above baseline VR use. In this study, there were episodes of cybersickness even in healthy volunteers during the 30-min VR session. These results have since prompted us to limit the use of VR to 10-min durations, followed by a 10-min rest break, in subsequent VR studies where we found that participants reported 100% tolerability.^21^

### Limitations

We note the contrast between positive qualitative feedback on the VR-delivered version and negative feedback on the PC Monitor-delivered version but were unable to observe quantitative differences in satisfaction between groups. We believe this is due to a strong ceiling effect, as exhibited by the clustering of scores in the upper interquartile range. Reasons for participants to score highly may be due to various reasons unrelated to the content but could be due to social desirability bias or deference.^22,23^ This study thus provides information that VR is a preferred mode of delivery, but due to this limitation, a more robust satisfaction questionnaire is required to confirm this.

Another limitation is the use of healthy volunteers, which we justify as our primary outcome was the impact of immersiveness on knowledge retrieval. Nonetheless, when used as intended in patients with cancer, multiple confounders would exist: presence of pain, medications affecting cognition, need for invasive medical procedures, and chemotherapy.

## Conclusion

This validation study for a custom-made VR program has been helpful by providing extra information for a follow-up study. In particular, feasibility and acceptability of VR will be improved by reducing the VR exposure from 30 min. Future trials recruiting patients with cancer, who are at even higher risk of cybersickness, would benefit from a protocol that schedules shorter, 10-min VR sessions with 10-min breaks. Knowledge retrieval was not shown to be superior for VR-based therapy versus PC monitor-based, but the positive feedback for the former combined with negative feedback on the latter supports future use of VR-based therapy.
